# “Pomacytosis”—Semi-extracellular phagocytosis of cyanobacteria by the smallest marine algae

**DOI:** 10.1371/journal.pbio.2003502

**Published:** 2018-01-05

**Authors:** Nina A. Kamennaya, Gabrielle Kennaway, Bernhard M. Fuchs, Mikhail V. Zubkov

**Affiliations:** 1 Ocean Biogeochemistry & Ecosystems Research Group, National Oceanography Centre, Southampton, United Kingdom; 2 Imaging and Analysis Centre, Science Facilities, Natural History Museum, London, United Kingdom; 3 Max-Planck Institute for Marine Microbiology, Bremen, Germany; Princeton University, United States of America

## Abstract

The smallest algae, less than 3 μm in diameter, are the most abundant eukaryotes of the World Ocean. Their feeding on planktonic bacteria of similar size is globally important but physically enigmatic. Tiny algal cells tightly packed with the voluminous chloroplasts, nucleus, and mitochondria appear to have insufficient organelle-free space for prey internalization. Here, we present the first direct observations of how the 1.3-μm algae, which are only 1.6 times bigger in diameter than their prey, hold individual *Prochlorococcus* cells in their open hemispheric cytostomes. We explain this semi-extracellular phagocytosis by the cell size limitation of the predatory alga, identified as the *Braarudosphaera* haptophyte with a nitrogen (N_2_)–fixing endosymbiont. Because the observed semi-extracellular phagocytosis differs from all other types of protistan phagocytosis, we propose to name it “pomacytosis” (from the Greek πώμα for “plug”).

## Author summary

The global significance of microorganisms is a consequence of their astronomical numbers. This is certainly true for the smallest planktonic algae on earth, which are less than 3 μm in diameter and the most numerous eukaryotic organisms of the oceans. Contrary to the general belief that algae use only sunlight and dissolved mineral nutrients to grow, these microscopic plants consume large numbers of bacteria. Their acting as mini-predators on bacteria of nearly their own size is hard to imagine. A tiny algal cell is cramped with organelles—such as nucleus, mitochondria, chloroplasts—and there is simply no space inside this cell to engulf a large bacterium in the usual manner. To find out how the 1.3-μm–diameter haptophyte algae feed, we studied them using high-resolution electron microscopy. We found that prey handling by the alga differs from all other types of cell feeding. We showed that this alga holds the 0.8-μm–diameter prey in the open cytostome (cell mouth) and that, from among planktonic bacteria, the alga apparently selects a ball-shaped *Prochlorococcus* (an abundant cyanobacteria responsible for most of global photosynthesis) that tightly fits into the open cytostome like a plug. Instead of full prey digestion, we observed that the alga leaves behind the doughnut-shaped carcass of the prey. We conclude that such unusual feeding, which we call “pomacytosis”, of this tiny predatory alga is caused primarily by the space limitation inside its cell.

## Introduction

In conventional phagocytosis, the caught prey is internalized, i.e., enclosed by a phagocytic membrane inside the predator cell to form a food vacuole, within which prey is digested and its contents are absorbed through the vacuole membrane [1]. Apart from secure isolation of the prey from the environment, full closure of the food vacuole benefits the predator in a number of ways. The fully closed vacuole allows the predator to pump excess water to reduce the vacuole volume, to adjust pH inside the vacuole to facilitate prey digestion by lytic enzymes, and to contain lysed prey for efficient nutrient assimilation. Only refractory prey material, e.g., moieties of cell wall, is egested when the closed food vacuole finally fuses back with the plasma membrane [2]. Thus, conventional phagocytosis of internalized prey requires enzymes, microfilament, microtubule, and membrane investments and can be limited by the predator size [3].

Phagocytosis of prey of similar size or bigger is difficult but achievable for protists. For example, some dinoflagellates use a feeding tube to inject lytic enzymes into prey and to extract digested prey contents [4,5]. Other dinoflagellates and several haptophytes form extracellular, yet closed, food vacuoles [6–8]. Such extensive extracellular vacuoles can only be completed by large predatory cells that can produce and stock sufficient amounts of the required investments. Compared to extracellular phagocytosis, internalization of similar-sized prey requires from the predator fewer investments but sufficient intracellular space free from organelles. In protists, the nucleus, mitochondria, and chloroplasts (the latter in algae) can vary in size, but these organelles cannot be smaller than a certain minimal volume. Owing to the presence of such “nonscalable” organelles [9], the intracellular volume available for investment storage and prey internalization shrinks as a power function of the predator cell size. Consequently, small protists may be unable to internalize (conventionally phagocytose) similar-sized prey. To test that, we focused on feeding of the smallest algae (<3 μm in diameter), whose chloroplast-packed cells in addition to the nucleus and mitochondria should have the minimal organelle-free space among free-living protists.

According to our morphometric estimates, organelles occupy approximately 70% of a haptophyte alga with a cell volume of 2.8 ± 0.8 μm^3^ (*n* = 10; S1 Fig). Even after taking into account scalable but vital cell components, e.g., endoplasmic reticulum rich in ribosomes and enzymes, both the haptophyte alga as well as the smallest known prasinophyte alga with a cell volume of 1.1 to 5.7 μm^3^ [10] are still capable of internalizing a bacterial cell of 0.1 to 0.3 μm^3^ [11]. This is in agreement with the substantial indirect experimental evidence that, despite their diminutive size, the smallest (1- to 3-μm diameter) algae are the main predators of bacterioplankton in the open ocean [12,13]. However, because of insufficient resolution of optical microscopy, phagocytosis by these algae could only be inferred [14].

In order to find out how algae less than 3 μm in size phagocytose similar-sized bacteria, we chose to study the smallest oceanic picoeukaryotic algae, plastidic eukaryote small (PES), separated from other protists and bacteria living in seawater by flow cytometry. Using high-resolution electron microscopy to observe fine cellular details of the sorted algae, we found that their semi-extracellular bacterial phagocytosis—“pomacytosis”—differs from all other types of phagocytosis.

## Results

Low concentrations of bacterioplankton and PES (6 × 10^5^ cells ml^-1^ and 4 × 10^2^ cells ml^-1^, respectively; S2 Fig) in the studied region of the Eastern subtropical North Atlantic Ocean were typical for open ocean waters [11,13].

**Fig 1:**
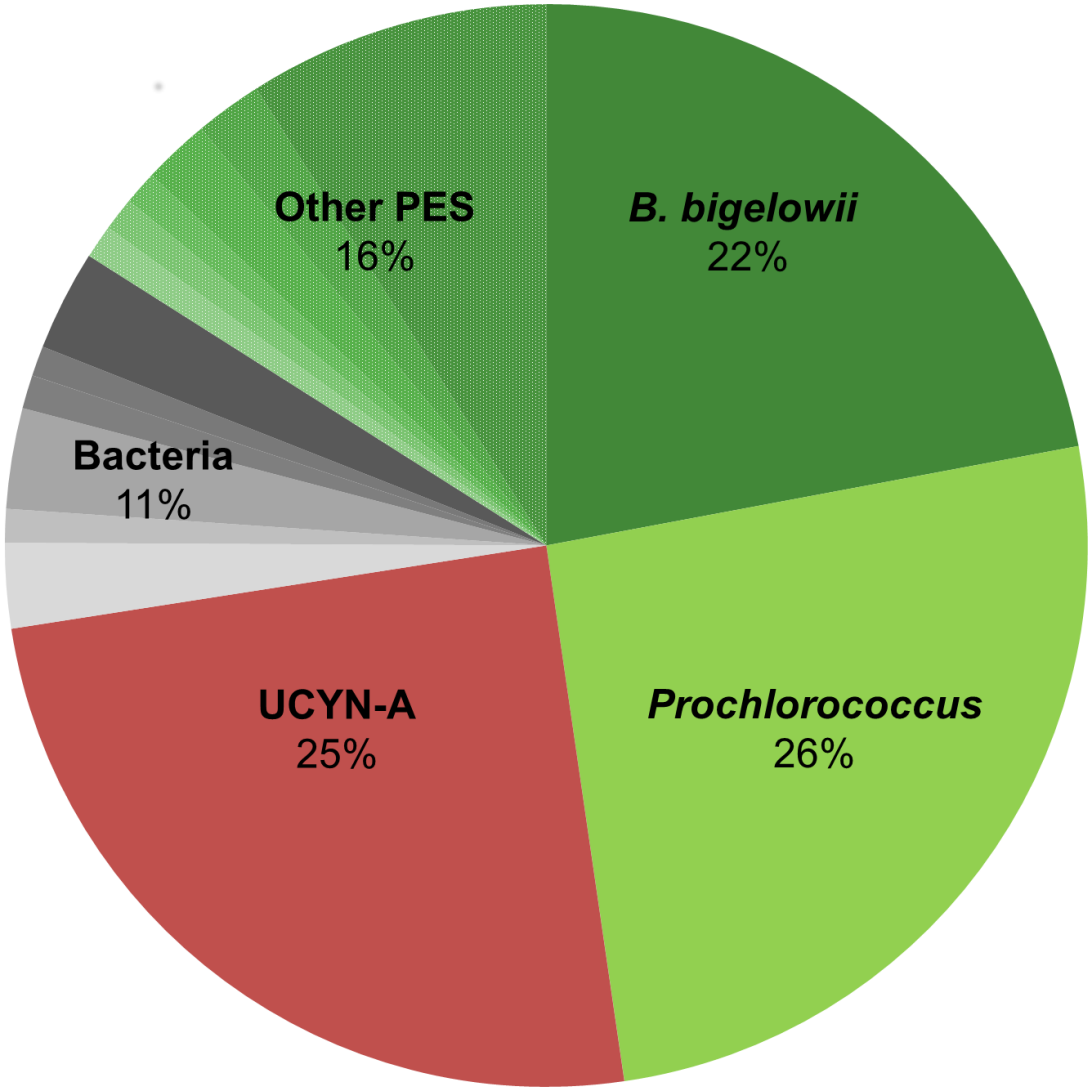
The diversity of 16S rRNA gene amplified from the flow-sorted PES cells. The 16S rRNA gene-based semiquantitative analysis identified three major constituents—the *Prochlorococcus*, UCYN-A cyanobacteria, and the haptophyte *Braarudosphaera bigelowii*. The remaining reads represented heterotrophic bacteria (deltaproteobacteria, 3%; Actinobacteria, 3%; Pseudomonadales, 1%; Rickettsiales, 1%; and others) and 10 other genera of small eukaryotic algae (*Ochromonas*, 9%; *Pelagomonas*, 2%; *Triparma*, 2%; *Imantonia*, 1%; *Chrysochromulina*, 1%; *Rhizochromulina*, 1%; and others). PES, plastidic eukaryote small; UCYN-A, unicellular diazotrophic cyanobacteria group A.

The main PES population was well defined by flow cytometry and selected for sorting (S3 Fig). High-throughput barcoding analysis of flow-sorted PES cells (S3 Fig) yielded 10,416 high-quality (≥300 nt) 16S rRNA gene reads and identified the dominant taxa: 51% of the amplicons were sequences of cyanobacteria, composed of *Prochlorococcus* (26%) and unicellular diazotrophic cyanobacteria group A (UCYN-A; 25%), and 38% of the amplicons were chloroplast sequences, the majority of which (58%) belonged to the Braarudosphaeraceae, a coccolithophore family of the Haptophyta (Fig 1). The remaining chloroplast sequences belonged to 10 other types of small algae, each of which represented only a minor fraction of the PES cells (Fig 1). The negligible number of sequences of SAR11 alphaproteobacteria (Rickettsidae)—the most abundant bacteria in the samples (thus the most probable by-sorted cells)—validated the high purity of PES sorting.

Analyses of nearly full-length ribosomal gene sequences confirmed the phylogenetic affiliation obtained with shorter amplicons. Full-length sequences of the 16S rRNA gene of *Prochlorococcus* and UCYN-A were 99% identical to high light–adapted *Prochlorococcus marinus* strain MIT9301 and 100% identical to the *Candidatus* Atelocyanobacterium thalassa isolate a long-term oligotrophic habitat assessment (ALOHA) [15], respectively. The 18S rRNA gene sequence was 99% identical to a calcifying *Braarudosphaera bigelowii* isolate TMRscBb7 [16] (S4 Fig) and to a small noncalcifying alga collected from oligotrophic waters of the South East Pacific Ocean (S4 Fig) [17], confirming the chloroplast 16S rRNA gene-based identification.

Scanning and transmission electron microscopy (SEM and TEM, respectively) showed no curved rod-shaped cells of the most abundant SAR11 bacteria (S5A Fig) among flow-sorted PES cells. The absence of by-sorted SAR11 bacteria reaffirmed the high sorting purity. The majority (95%) of the imaged PES cells (185 out of 195) were ball-shaped small cells with an estimated diameter of 1.3 ± 0.22 μm (*n* = 33, size corrected for 30% linear cell shrinkage during sample dehydration [18]). Some of them bore organic, noncalcified scales (S6 Fig). These morphotypes represent coccolythophore life cycle stages found in nutrient-poor waters [19–21]. Among the sorted PES cells, there were no cells with external mineral investments, i.e., pentagonal-shape liths characteristic of *Braarudosphaera* species found in nutrient-replete waters [22]. A few morphologically different cells (10 out of 195 examined cells) had one or two well-preserved flagella (S7 Fig) that ruled out the artificial loss of external investments by the dominant alga.

**Fig 2:**
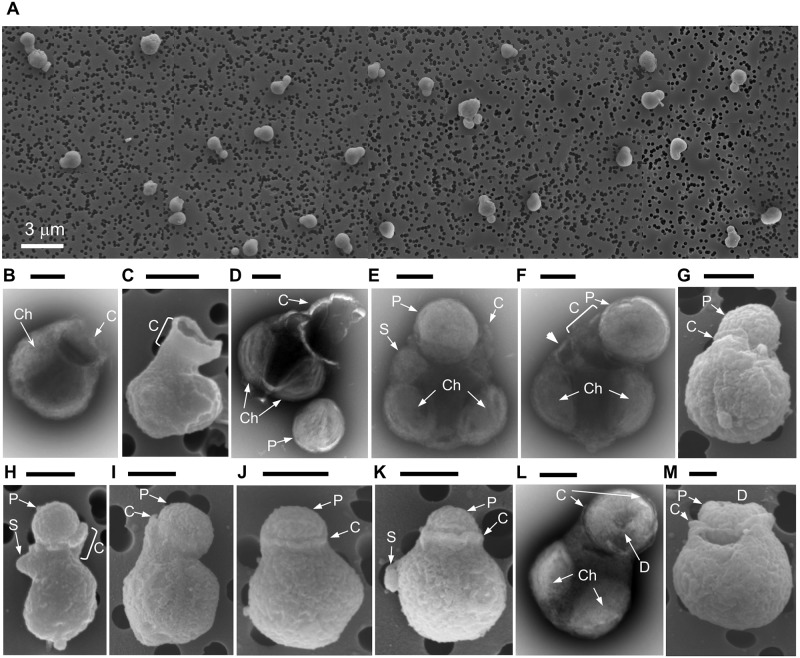
Electron microscopy observations of selective feeding by naked *Braarudosphaera* algae on *Prochlorococcus* cyanobacteria. (A) The collage depicting that the sorted smallest picoeukaryotic algae were dominated by a single morphological type of cells molecularly identified as *B*. *bigelowii* JC142. Note that the majority of the *B*. *bigelowii* JC142 cells are associated with prey cells molecularly identified as *Prochlorococcus*. (B–M) Representative micrographs that depict *B*. *bigelowii* cells with *Prochlorococcus* prey (E–M, groups a and b) or with lost *Prochlorococcus* (B–D, group c). In E–H, less than half of the captured *Prochlorococcus* cell is covered with a cytostome (group a), and in I–M, more than half of the *Prochlorococcus* cell is embraced (group b). Scale bar = 0.5 μm. The figure combines the SEM (A, C, G, H, I, J, K, and M) and TEM (B, D, E, F, and L) micrographs. C, cytostome; Ch, chloroplast; D, doughnut-shape deformation of the consumed *Prochlorococcus* cell; JC142, the Royal Research Ship “James Cook” cruise number 142; P, *Prochlorococcus*; S, UCYN-A cyanobiont; SEM, scanning electron microscopy; TEM, transmission electron microscopy.

Out of 185 cells of the dominant alga, 155 (84%) were associated with smaller coccoid cells 0.81 ± 0.08 μm (*n* = 10, size corrected for 30% linear cell shrinkage during cell dehydration) in diameter (Fig 2). An additional intracellular body of the dominant algal cells was observed using TEM (Fig 2E). The 0.47 ± 0.05 μm (*n* = 4) diameter body occupied a particular location at the cell periphery next to one of the two chloroplasts. When the body was absent, a rupture in the algal cell wall was observed (Fig 2F, thick arrow; S5B Fig), confirming that the body was intracellular but could be lost under mechanical stress caused by sorting PES cells directly on TEM grids. A similar intracellular “spheroid body” in *B*. *bigelowii* isolate TMRscBb7 was identified as an obligate N_2_-fixing UCYN-A endosymbiont [16]—cyanobiont. Contrary to the cyanobiont, the molecularly identified *Prochlorococcus* associated with PES is a free-living planktonic cyanobacterium that was numerous in the studied seawater (1.7 × 10^5^ cells ml^-1^).

Synthesising the above evidence, we concluded that the UCYN-A amplicon derived from the “intracellular body” and the *Prochlorococcus* amplicons represented the extracellular cocci attached to the PES cells. We interpreted the latter association as phagocytosis of *Prochlorococcus* by the naked haptophyte (hereafter referred to as *B*. *bigelowii* JC142). We assigned the observed *B*. *bigelowii* cells to two major groups and one minor group, as follows: (a) alga with an associated *Prochlorococcus*, of which less than 50% cell surface is inside the cytostome (49%); (b) alga with an associated *Prochlorococcus*, of which more than 50% cell surface is inside the cytostome (35%); and (c) alga with a cytostome but without prey (16%) (Fig 2).

The cytostome is most likely used for shape- (and possibly surface-)selective prey recognition and capture. In support of the notion of selection, neither molecular nor microscopic evidence suggested that *B*. *bigelowii* JC142 fed on SAR11 alphaproteobacteria—the most abundant free-living bacteria in the studied seawater (2.8 × 10^5^ cells ml^-1^; S2 Fig). The algae preferred to feed on less abundant *Prochlorococcus* (1.7 × 10^5^ cells ml^-1^), which made up only 27% of total bacterioplankton in the seawater (6 × 10^5^ cells ml^-1^; S2 Fig), i.e., the haptophyte selected on average one out of four encountered free-living bacterial cells.

Because of high-purity PES sorting, the individual *Prochlorococcus* observed by SEM were not by-sorted cells but were in fact cells detached from the haptophytes during sorting (e.g., Fig 2D). Both intact and doughnut-shaped, deformed *Prochlorococcus* cells were observed (S5 Fig). The intact, spherical *Prochlorococcus* (14 observed cells) were probably at the start of pomacytosis, while the doughnut-shaped *Prochlorococcus* with a central small spot of depressed surface area (23 cells) were at the end of pomacytosis (S5 Fig). Similarly, deformed *Prochlorococcus* cells were observed by SEM and TEM (Fig 2L and 2M), affirming that the deformation was a result of pomacytosis rather than an artefact of SEM sample preparation.

High-power TEM revealed that in the groups (a) and (b), the prey *Prochlorococcus* cell is fitted into a semicircular cytostomic depression, which—according to SEM—is in reality hemispherical and is anchored in the cytostome between the two algal chloroplasts (Fig 2C and 2E). In all 155 specimens observed with the *Prochlorococcus* cell, the latter remains at least partially free of the algal cytostome membrane (Fig 2, S8 Fig). To our knowledge, this is the first observation of semi-extracellular phagocytosis of prey by a protist using a partially opened cytostome.

## Discussion

The *B*. *bigelowii* JC142 is the smallest haptophyte that was directly observed to phagocytose free-living bacteria. However, *B*. *bigelowii* ability to internalize the selected bacterium is evolutionary evidenced—its cyanobiont is of a phagocytic origin. The intracellular UCYN-A symbiont cell in *B*. *bigelowii* isolate TMRscBb7 is surrounded by a food vacuole membrane [16]. The presence of the UCYN-A cyanobiont further reduces the intracellular space of *B*. *bigelowii* available for prey internalization. The size of the UCYN-A symbiont of *B*. *bigelowii* JC142 is at the lowest end of the reported UCYN-A size range [15,16,23,24]. The cyanobiont occupies less than 5% of the estimated volume of the *B*. *bigelowii* JC142 cell, while the *Prochlorococcus* prey measures more than 20% of the algal volume. Perhaps the choice between conventional phagocytosis and pomacytosis depends on the size ratio between the alga and its prey. In order to overcome its space limitation, 1.3-μm *B*. *bigelowii* JC142 cell, pomacytoses 0.8-μm *Prochlorococcus* instead of using whole-cell phagocytosis.

Selective feeding of *B*. *bilgelowii* JC142 on *Prochlorococcus* implies that, despite the internal supply of fixed nitrogen by the UCYN-A cyanobiont—or perhaps owing to this supply as well as to metabolic demands of the symbiont—the haptophyte could be limited in other main inorganic nutrients [25], e.g., phosphorus and iron. However, this limitation is unlikely because *B*. *bigelowii* JC142 was collected in the Eastern subtropical North Atlantic Ocean fertilised by aeolian dust from the Saharan desert. Consequently, the surface waters in the studied area are enriched in phosphate and iron [26] but are poor in nitrogen salts [27]—the environment that facilitates the growth of N_2_-fixing photoautotrophs. Instead of photoautotrophy, *B*. *bigelowii* JC142 cells unconstrained by inorganic nutrients, including nitrogen (fixed by its cyanobiont), pomacytose *Prochlorococcus*. Therefore, the main nutrient the haptophytes gain from *Prochlorococcus* prey is, perhaps, fixed carbon.

*B*. *bigelowii* may require fixed carbon because it has the cyanobiont. The UCYN-A cyanobiont lost its photosystem II complex (PSII) but retained its photosystem I (PSI) [28] to use light energy to fix N_2_. In return for the shared fixed nitrogen, the *Braarudosphaera* host should share its fixed carbon with the cyanobiont [15,29]. Furthermore, to minimize inhibition of the cyanobiont N_2_ fixation, a *B*. *bigelowii* cell needs to keep its intracellularly dissolved O_2_ concentration low. Large host cells, e.g., *Rhizosolenia* and *Rhopalodia* diatoms, do that by spatially segregating their chloroplasts from N_2_-fixing cyanobionts within their cells [30]. In the 1.3-μm *B*. *bigelowii* JC142 cell (Fig 2E), O_2_ produced by the adjacent chloroplast could directly inhibit N_2_ fixation by the cyanobiont, and the haptophyte needs to reduce [29] if not to halt photosynthesis by its own chloroplasts. Consequently, both the host and cyanobiont become starved of fixed carbon and require its alternative, external source. In order to acquire that fixed carbon, *B*. *bigelowii* JC142 selectively pomacytose free-living *Prochlorococcus* cyanobacteria.

Based on our observations (Figs 1 and 2), we suggest interpreting the reported association between the “unknown structure” and UCYN-A–bearing haptophyte (Fig 6 in [29]) as *Prochlorococcus* cell pomacytosed by the haptophyte. Low CO_2_ fixation by the haptophyte chloroplasts compared with high CO_2_ fixation by the “unknown structure”—*Prochlorococcus* (Fig 6 in [29])—supports our suggestion that the *Braarudosphaera* could acquire fixed carbon from its prey rather than from its own chloroplasts. Perhaps, because a CO_2_-fixing *Prochlorococcus* cell also produces O_2_, the *B*. *bigelowii* JC142 cell does not internalize it. Instead, live *Prochlorococcus* is kept segregated from the O_2_-sensitive cyanobiont, and the haptophyte keeps the cytostome semi-open to allow O_2_ dissipation. Therefore, pomacytosed *Prochlorococcus* could be viewed as a temporary chloroplast substitute.

Conventional phagocytosis is a relatively quick process that usually takes seconds (e.g., [8]), and one seldom observes a protist predator in the process of internalizing prey. Because the majority of the *B*. *bigelowii* JC142 collected during six-hour sampling was in a process of feeding (84% held prey), pomacytosis should be a slow process that takes hours. The absence of internalized *Prochlorococcus* cells and nearly 1:1 ratio between pomacytosed *Prochlorococcus* with more than half cell surface exposed (group [a]) and with less than half cell surface exposed (group [b]) suggest that the haptophyte controls exposure of the prey cell to seawater. During slow pomacytosis, the predator could gain extra benefit from the prey that fixes CO_2_ and takes up nutrients through the cell wall exposed to seawater (Fig 2E–2K). Unlike conventionally phagocyting cells, the pomacyting *B*. *bigelowii* JC142 detained *Prochlorococcus* in their cytostome without full internalization, perhaps; harvested fixed carbon released by prey; and egested the deformed, spent prey without full digestion (S5 and S8 Figs).

Therefore, a combination of intracellular space limitation (primarily) and physiological requirements of the tiny predatory alga (secondarily) leads to semi-extracellular phagocytosis of selected prey.

## Materials and methods

### Ethics statement

This is an oceanographic study carried out in the international waters. This research does not require special permission.

### Sampling collection and sorting strategies

The study was carried out in the Eastern subtropical North Atlantic Ocean (23° 37′ N 20° 43′ W) on board the Royal Research Ship “James Cook” during the research cruise JC142 from November to December 2016. Seawater samples from 25 m (a representative depth of the surface mixed layer) were collected using a rosette of 20-l Niskin bottles mounted on a conductivity-temperature-depth (CTD) profiler. All plastic- and glass-ware for handling seawater was prewashed with 10% HCl and rinsed with sampled seawater.

Concentrations of total bacterioplankton, including *Prochlorococcus* and SAR11, the latter as a population of cells with low nucleic acid content [31], were determined by flow cytometry. Routinely, samples were fixed with 1% (w/v) paraformaldehyde (PFA) final concentration, stained with SYBR Green I DNA dye [11,32], and analysed with the custom-modified FACSort instrument (Becton Dickinson, Oxford, UK) equipped with the blue diode laser (488 nm, 50 mW; Quantum Analysis, Munster, Germany) using the CellQuest software.

For determining concentrations of PES and *Synechococcus* and for cross-referencing microbial populations in the concentrated samples (used for flow sorting), seawater samples were fixed with 2% PFA, stained with 0.1 μg ml^-1^ Hoechst 33342 (final concentration), and analysed with the custom-built MoFlo XDP instrument (Beckman-Coulter, High Wycombe, UK) (S2 Fig) using the Summit 5.4 software. The first UV diode laser (355 nm, 100 mW; JDSU, CY355-100, Thailand) and the second blue diode laser (488 nm, 240 mW; Cobolt, Solna, Sweden) were aligned through the first and third pinhole, respectively. Shallow angle light scatter (forward scatter [FSC]) of the UV light was detected using the 351 ± 5–nm optical filter and the H957-18 photomultiplier (Hamamatsu, Japan). More sensitive H957-27 photomultipliers (Hamamatsu) were used for detecting particle fluorescence at four wavelengths (457 ± 25 nm, 530 ± 20 nm, 580 ± 15 nm, >643 nm) and the three wavelengths (505–550 nm, 580 ± 15 nm, 670 ± 15 nm) excited by the first and second laser, respectively.

A reference mixture of yellow-green (505/515 nm) 0.5-μm beads (Life Technologies, Eugene, Oregon, US) and multifluorescence 1.0-μm beads (Fluoresbrite Microparticles, Polysciences, Warrington, Pennsylvania, US) were used as an internal standard for both fluorescence and flow rates. The absolute concentration of beads in the stock solution was determined using syringe pump flow cytometry [33].

For flow sorting, microbes were gravity concentrated approximately 10^3^-fold using sterile 0.2-μm pore size Sterivex filter units (Millipore, Watford, UK) attached directly to Niskin bottles. For molecular identification, concentrated microbial samples were fixed with Lugol iodine solution [34] and stored at +4°C before being flow sorted within 48 hours. Samples were discoloured with thiosulfate [34] and stained with Hoechst 33342 prior to sorting. For electron microscopy analyses concentrated samples were fixed with 2% PFA and stained with Hoechst 33342 prior to sorting. The same dominant distinct population of the smallest picoeukaryotic algae—PES—was flow sorted with the MoFlo XDP instrument (S3 Fig) using the Summit 5.4 software. The instrument was optically aligned, and its sorting purity and recovery were optimised using blue (350/440 nm) 1.0-μm beads (Life Technologies). Only PES cells gated by both gates (S3B, S3C, S3E and S3F Fig) were sorted. Purity of sorted PES cells was validated by the molecular and electron microscopy analyses.

### Microscopy

For TEM analyses, 1 × 10^3^ to 2 × 10^3^ target PES cells were flow sorted directly on formvar/carbon–covered 200 mesh copper grids (Agar Scientific, Stansted, UK) stained with 2% w/v Gadolinium (aqueous solution), rinsed with pure deionized water, and stored in a desiccator for analysis ashore. The grids were examined at 200 keV with the Jeol 2011 LaB6 TEM instrument fitted with a Gatan UltraScan 1000 camera at the University of Warwick’s Research Technology Platform in Advanced Bioimaging in the United Kingdom.

For SEM analyses, 20 × 10^3^ target cells were flow sorted into sterile 1.5-ml microcentrifuge tubes containing aqueous solution of 1% glutaraldehyde (Electron Microscopy Sciences). The tubes were stored at 4°C and brought ashore. The sorted cells were collected onto 0.2-μm pore size, 13-mm polycarbonate filters under low vacuum, dehydrated in the ethanol series, and critical point dried using 99.9% hexamethyldisilazane (Sigma-Aldrich). The dehydrated filters were stored in a desiccator at room temperature. Prior to SEM analyses, the filters were sputtered with Au/Pd (3:2) to a thickness of 10 nm using the High-Resolution (208hr) Sputter Coater coupled with the MTM20 film thickness controller (Cressington). The filters were examined with the high-resolution SEM UltraPlus instrument (Zeiss Gemini) at 5 keV using the secondary electron detector at the Imaging and Analysis Centre of the Natural History Museum in London, UK.

Cell dimensions were measured on both TEM and SEM micrographs using the ImageJ software [35]. The values obtained from the SEM micrographs were corrected to account for approximately 30% cell shrinkage [18]. Average cell volumes were calculated assuming a ball or spheroid shape of algal cells (4/3πa^2^b), a spherical segment for chloroplasts (πh^2^[b-1/3h]), an ellipsoid for a nucleus (4/3π[a-h]^2^b), and half of this ellipsoid for a mitochondrion (S1 Fig).

### Molecular identification

For molecular analyses, 20 × 10^3^ to 50 × 10^3^ PES cells were flow sorted into sterile 1.5-ml microcentrifuge tubes. An aliquot of 2 μl containing approximately 2 × 10^3^ cells was added into a 0.2-ml PCR tube containing 30 μl of Q5 High Fidelity Master Mix (New England BioLabs) complemented with primers and nuclease-free water (Ambion). For full-length 16S or 18S rRNA gene amplification, we used 27f/1492r [36] or 63f/1818r [37] primers with annealing temperature of 59°C. The amplicons were added with A-tails (OneTaq DNA polymerase, New England BioLabs), ligated to the pGEM T-Easy vector (Promega), and transformed into the NEB 5-alpha competent *Escherichia coli* cells (New England BioLabs). Plasmids from the positive colonies were sequenced with T7 and SP6 primers to cover the full amplicon length. The 18S rRNA gene sequences were aligned with 18 reference sequences of haptophytes (1,400 positions), and phylogenetic relationships for the dataset were calculated with MrBayes software [38].

For a massively parallel sequencing, hyper variable regions V3–V4 (490 bp) were amplified by PCR using S-D-Bact-0341-b-S-17 and S-D-Bact-0785-a-A-21 primers [39]. The forward primer included the PGM barcode adapter (Ion Xpres Barcode Adapters 1–96 Kit, ThermoFisher Scientific), and both primers were tailed with the Ion Torrent sequencing adapters to allow direct downstream multiplexed sequencing. Following amplification, PCR products of approximately 490 bp were gel purified with NucleoSpin Gel and PCR Cleanup kit (Macherey-Nagel), and 1.5 ng of the product was used for template preparation with the Ion Torrent OneTouch System (ThermoFisher Scientific). The templates were sequenced on an Ion Torrent PGM sequencer (ThermoFisher Scientific) using the Hi-Q sequencing chemistry.

After sequencing, the individual sequence reads were first quality trimmed using the Ion Torrent software suite and then further processed using the bioinformatics pipeline of the Silva NGS project [40]. This involved quality controls for sequence length (≥300 bp) and the presence of ambiguities (<2%) and homopolymers (<2%). The remaining reads were split into individual sample FASTA files using mothur [41] and aligned against the SSU rRNA seed of the SILVA database release 119. The classification was done by a local BLAST search against the SILVA SSU Ref 115 nonredundant (NR) database using BLAST 2.2.22+ with standard settings. The analysis gave (semi)quantitative information (number of individual reads representing in a taxonomic pool) on the composition of the original PCR amplicon pool [39]. The classification of plastidic SSU rRNA sequence reads was done by nucleotide BLAST search against the NR database at the National Center for Biotechnology Information (NCBI; www.ncbi.nlm.nih.gov).

## Supporting information

S1 Fig A set of the representative smallest algal cells used for the initial morphometric estimation of cellular volume available for conventional phagocytosis. (A–F) The six examples of the smallest algal cells flow sorted from seawater samples collected at the bottom of the mixed layer (100–120 m, approximately 5 m above the deep chlorophyll maximum) in the South Atlantic subtropical gyre during the Atlantic Meridional Transect cruise AMT24 in October 2014. (G) The derived schematic model of a typical algal cell with the marked morphometric parameters used to estimate the total cell volume and the volumes of cell organelles. chl, chloroplast; mit, mitochondrion; N, nucleus.
(TIF)

S2 Fig Flow cytometric signatures of Hoechst DNA–stained planktonic microbes analysed by the MoFlo instrument. (A) A density plot of shallow angle light scatter (FSC) versus tailed Hoechst DNA 530 ± 20–nm fluorescence showing the population of stained Bpl above the set threshold. (B) A density plot showing the populations of stained Bpl and of the smallest picoeukaryotic algae (PES) relative to the reference beads. (C) A density plot showing the populations of PES and *Syn* based on their Chl and PE autofluorescence, exited by the second laser. The *Pro* population is partially resolved because of extremely low Chl autofluorescence of their cells. (D) A density plot showing the populations of Bpl and PES, based on their DNA staining and extra Chl autofluorescence of the latter, exited by the first laser. Arrows and dotted-line polygons indicate populations of the analysed cells and clusters of reference beads: 0.5-μm yellow-green beads (0.5Bd), 1.0-μm multifluorescence beads (1.0Bd), and 1.0-μm blue beads (1.0UV). The 0.5Bd clusters were smeared because of low yellow-green bead fluorescence at 457 nm and 670 nm. Owing to >10^3^ higher cell numbers of Bpl compared with PES, the PES population is considerably less dense. A total of 2.2 × 10^6^ events were recorded, including 2.5 × 10^5^ Bpl, 2.7 × 10^3^ *Pro*, 10^3^ *Syn*, and 150 PES cells. Bpl, bacterioplankton; Chl, Chlorophyll; FSC, forward scatter; PE, Phycoerythrin; PES, plastidic eukaryote small; *Pro*, *Prochlorococcus*; *Syn*, *Synechococcus*.
(TIF)

S3 Fig Characteristic flow cytometric signatures of Hoechst DNA–stained planktonic microbes in concentrated samples flow sorted by the MoFlo instrument. Signatures of Lugol-fixed (left column) and PFA-fixed (right column) cells are compared. (A) and (D) Paired density plots of shallow angle light scatter (FSC) versus tailed Hoechst DNA 530 ± 20–nm fluorescence showing the populations of stained Bpl, of *Syn*, and of the smallest picoeukaryotic algae (PES) above the set threshold. (B) and (E) Paired density plots of FSC versus core Hoechst DNA 457 ± 25–nm fluorescence showing the Bpl, *Syn*, and PES populations. (C) and (F) Paired density plots of extra Chl autofluorescence >643 nm versus core Hoechst DNA 457 ± 25–nm fluorescence showing the Bpl and PES populations. Note on (C) that, although the Chl autofluorescence of PES was bleached by Lugol, the PES population remains separated from the Bpl population. Arrows indicate populations of Bpl, *Syn*, and PES. Only the PES cells gated by a pair of the dotted-line polygons on B–C and on E–F, respectively, were flow sorted. A total of 5 × 10^6^ events were recorded for both Lugol-fixed and PFA-fixed samples, of which 2.8 × 10^6^ and 3.2 × 10^6^ were Bpl cells, respectively; 1.7 × 10^3^ and 3.9 × 10^3^ were PES cells, respectively. The PES cell numbers indicate the number of sorted cells present in the paired gates. Bpl, bacterioplankton; Chl, Chlorophyll; FSC, forward scatter; PES, plastidic eukaryote small; PFA, paraformaldehyde; *Syn*, *Synechococcus*.
(TIF)

S4 Fig Phylogenetic affiliation of *B*. *bigelowii* JC142 flow sorted from the Eastern subtropical North Atlantic Ocean. The Bayesian inference phylogenetic tree of 18S rRNA gene sequences of *B*. *bigelowii* JC142 and selected cultured haptophytes, which shows close relationship between the *B*. *bigelowii* JC142 (Accession number MF185178) and *B*. *bigelowii* isolate TMRscBb7. The NCBI accession numbers of cultured haptophytes are given in parentheses. Posterior probabilities of the Bayesian inference analysis are represented with symbols: * = 1, # = 0.9, ° = 0.6. NCBI, National Center for Biotechnology Information.
(TIF)

S5 Fig Examples of electron microscopy micrographs of free-living SAR11 alphaproteobacteria, the lost UCYN-A cyanobiont of *B*. *bigelowii* JC142, morphologically intact and deformed *Prochlorococcus* cells. (A) SAR11 alphaproteobacterial cells are presented to compare their morphology with morphology of *Prochlorococcus* cells. (B) A 0.4 × 0.5–μm body recorded with TEM is most likely a UCYN-A cyanobiont of *B*. *bigelowii*, which was lost during deposition of flow-sorted algae onto a grid. (C–K) Ball-shaped intact *Prochlorococcus* cells (H–J, thick arrows) compared to *Prochlorococcus* cells with characteristic morphological deformations (thin arrows). In the deformed *Prochlorococcus* cells, note the depression(s), which transform the *Prochlorococcus* cells from a ball shape into a doughnut shape. The deformed *Prochlorococcus* cells were held by *B*. *bigelowii* JC142 and presumably separated from them during sorting and dehydration. Scale bar = 0.2 μm. TEM, transmission electron microscopy; UCYN-A, unicellular diazotrophic cyanobacteria group A.
(TIF)

S6 Fig The cumulative spectrum of SEM-coupled energy dispersive X-ray spectroscopy collected as a line across the *B*. *bigelowii* JC142 cell covered with visible extracellular investments. The collected spectrum has distinct peaks of C, N, and O of algal organic materials (polycarbonate support filter contributed only to the C signal) as well as peaks of Au, Pt, and Al originated from the sputtered Au-Pt coating and the aluminium sample stub. The extracellular scale-like investment (arrow) is not calcified because the spectrum showed no detectable Ca. Ca, calcium; SEM, scanning electron microscopy.
(TIF)

S7 Fig Representative SEM images of the flow-sorted smallest picoeukaryotic algae (PES), which were morphologically different from the dominant haptophyte *B*. *bigelowii* JC142. Out of 195 examined cells, only 10 cells had alternative morphology. Note isokont flagella with the distinct basal bodies and pointed tips. Scale bar = 0.5 μm. JC142, the Royal Research Ship “James Cook” cruise number 142; SEM, scanning electron microscopy; PES, plastidic eukaryote small.
(TIF)

S8 Fig Incomplete enclosure of the *Prochlorococcus* prey with a cytostome of *B*. *bigelowii* JC142 predator. Representative TEM (A) and SEM (B) micrographs show how the *Prochlorococcus* cell is embraced with the partially open cytostome. Arrows indicate the cytostome edge. Scale bar = 0.2 μm. Ch, chloroplast; P, *Prochlorococcus* prey; S, cyanobiont; SEM, scanning electron microscopy; TEM, transmission electron microscopy.
(TIF)

## Abbreviations

ALOHA: a long-term oligotrophic habitat assessment
CTD: conductivity-temperature-depth
FSC: forward scatter
JC142: the Royal Research Ship “James Cook” cruise number 142
NCBI: National Center for Biotechnology Information
NR: nonredundant
PES: plastidic eukaryote small
PFA: paraformaldehyde
PSI: photosystem I complex
PSII: photosystem II complex
SEM: scanning electron microscopy
TEM: transmission electron microscopy
UCYN-A: unicellular diazotrophic cyanobacteria group A
