# Supplementary figures and images for: “Pomacytosis”—Semi-extracellular phagocytosis of cyanobacteria by the smallest marine algae
Source: PLoS Biol. 2018 Jan 5;16(1):e2003502. doi: 10.1371/journal.pbio.2003502 (PMC5773223; doi:10.1371/journal.pbio.2003502)

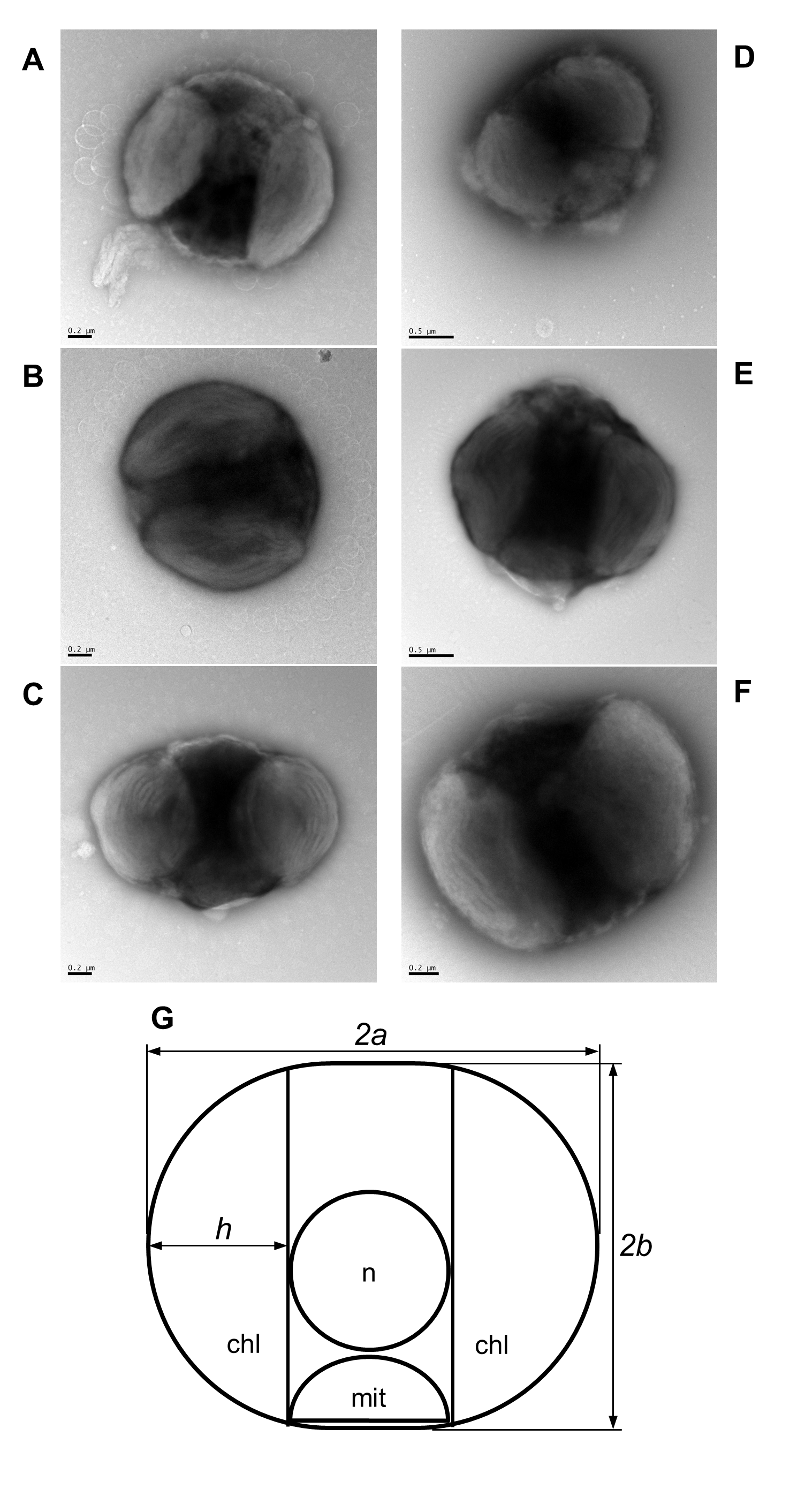

Supplement: S1 Fig — (A–F) The six examples of the smallest algal cells flow sorted from seawater samples collected at the bottom of the mixed layer (100–120 m, approximately 5 m above the deep chlorophyll maximum) in the South Atlantic subtropical gyre during the Atlantic Meridional Transect cruise AMT24 in October 2014. (G) The derived schematic model of a typical algal cell with the marked morphometric parameters used to estimate the total cell volume and the volumes of cell organelles. chl, chloroplast; mit, mitochondrion; N, nucleus. (TIF) [file pbio.2003502.s001.tif]

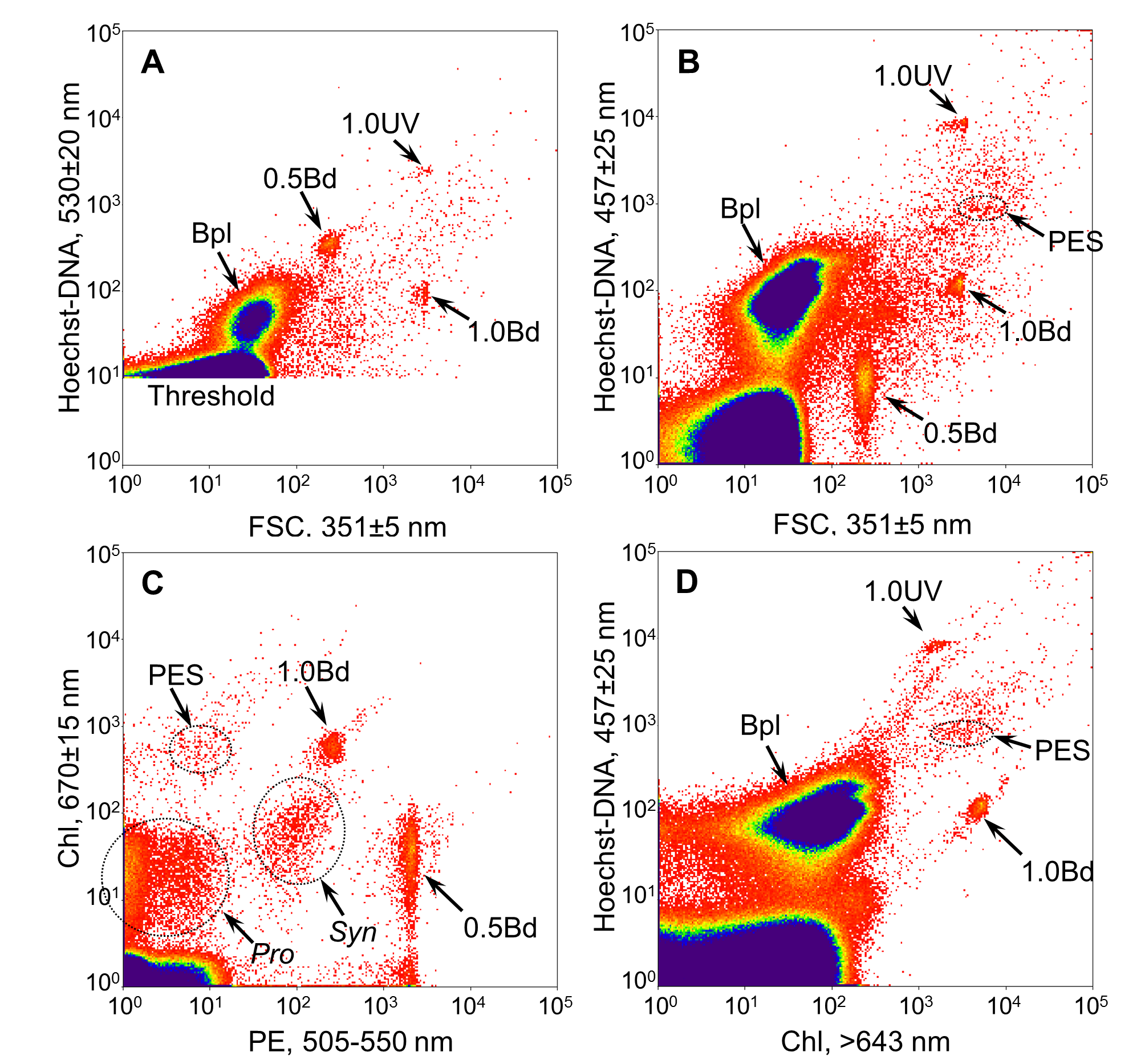

Supplement: S2 Fig — (A) A density plot of shallow angle light scatter (FSC) versus tailed Hoechst DNA 530 ± 20–nm fluorescence showing the population of stained Bpl above the set threshold. (B) A density plot showing the populations of stained Bpl and of the smallest picoeukaryotic algae (PES) relative to the reference beads. (C) A density plot showing the populations of PES and Syn based on their Chl and PE autofluorescence, exited by the second laser. The Pro population is partially resolved because of extremely low Chl autofluorescence of their cells. (D) A density plot showing the populations of Bpl and PES, based on their DNA staining and extra Chl autofluorescence of the latter, exited by the first laser. Arrows and dotted-line polygons indicate populations of the analysed cells and clusters of reference beads: 0.5-μm yellow-green beads (0.5Bd), 1.0-μm multifluorescence beads (1.0Bd), and 1.0-μm blue beads (1.0UV). The 0.5Bd clusters were smeared because of low yellow-green bead fluorescence at 457 nm and 670 nm. Owing to >103 higher cell numbers of Bpl compared with PES, the PES population is considerably less dense. A total of 2.2 × 10^6^ events were recorded, including 2.5 × 10^5^ Bpl, 2.7 × 10^3^ Pro, 103 Syn, and 150 PES cells. Bpl, bacterioplankton; Chl, Chlorophyll; FSC, forward scatter; PE, Phycoerythrin; PES, plastidic eukaryote small; Pro, Prochlorococcus; Syn, Synechococcus. (TIF) [file pbio.2003502.s002.tif]

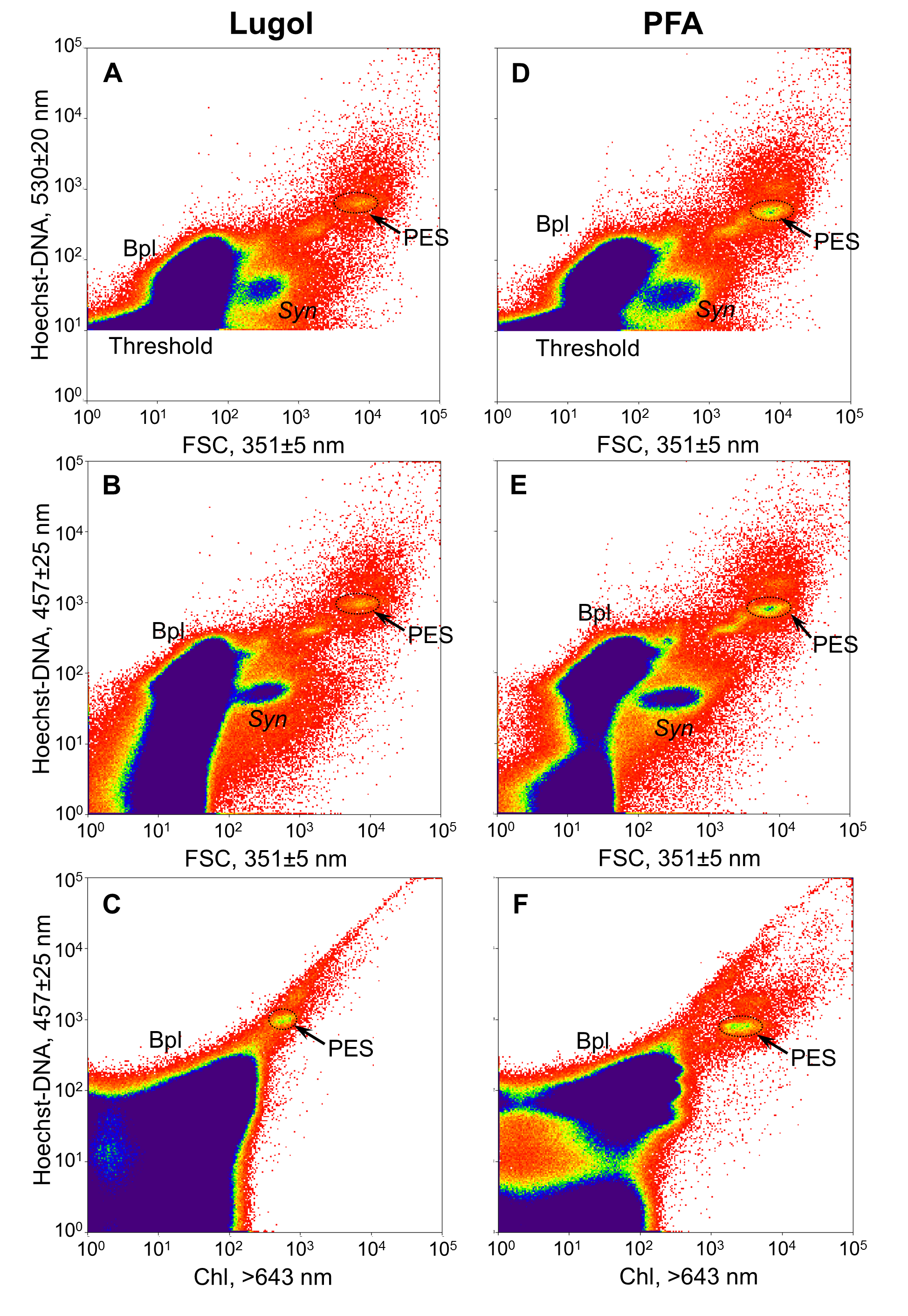

Supplement: S3 Fig — Signatures of Lugol-fixed (left column) and PFA-fixed (right column) cells are compared. (A) and (D) Paired density plots of shallow angle light scatter (FSC) versus tailed Hoechst DNA 530 ± 20–nm fluorescence showing the populations of stained Bpl, of Syn, and of the smallest picoeukaryotic algae (PES) above the set threshold. (B) and (E) Paired density plots of FSC versus core Hoechst DNA 457 ± 25–nm fluorescence showing the Bpl, Syn, and PES populations. (C) and (F) Paired density plots of extra Chl autofluorescence >643 nm versus core Hoechst DNA 457 ± 25–nm fluorescence showing the Bpl and PES populations. Note on (C) that, although the Chl autofluorescence of PES was bleached by Lugol, the PES population remains separated from the Bpl population. Arrows indicate populations of Bpl, Syn, and PES. Only the PES cells gated by a pair of the dotted-line polygons on B–C and on E–F, respectively, were flow sorted. A total of 5 × 10^6^ events were recorded for both Lugol-fixed and PFA-fixed samples, of which 2.8 × 10^6^ and 3.2 × 10^6^ were Bpl cells, respectively; 1.7 × 10^3^ and 3.9 × 10^3^ were PES cells, respectively. The PES cell numbers indicate the number of sorted cells present in the paired gates. Bpl, bacterioplankton; Chl, Chlorophyll; FSC, forward scatter; PES, plastidic eukaryote small; PFA, paraformaldehyde; Syn, Synechococcus. (TIF) [file pbio.2003502.s003.tif]

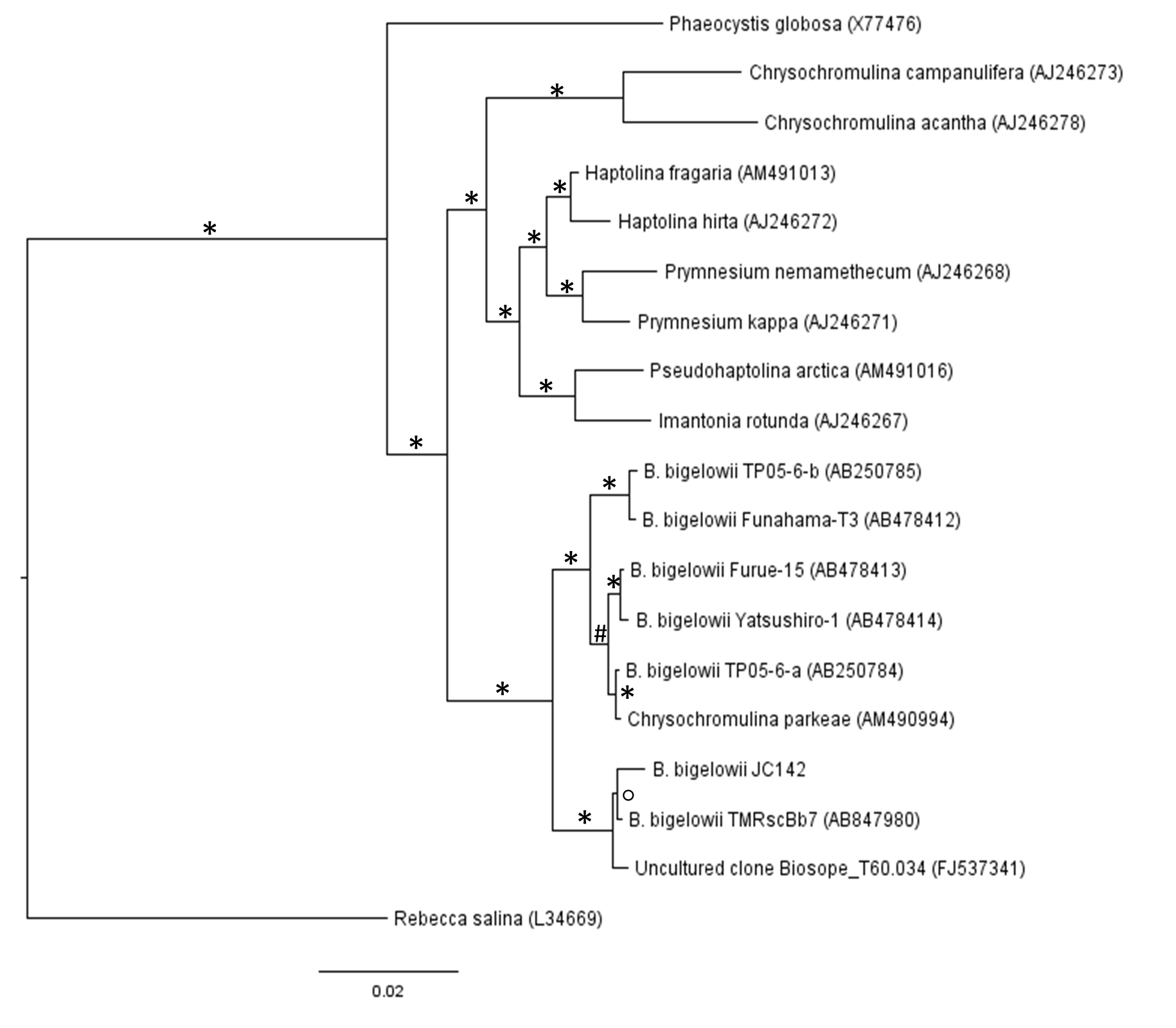

Supplement: S4 Fig — The Bayesian inference phylogenetic tree of 18S rRNA gene sequences of B. bigelowii JC142 and selected cultured haptophytes, which shows close relationship between the B. bigelowii JC142 (Accession number MF185178) and B. bigelowii isolate TMRscBb7. The NCBI accession numbers of cultured haptophytes are given in parentheses. Posterior probabilities of the Bayesian inference analysis are represented with symbols: * = 1, # = 0.9, ° = 0.6. NCBI, National Center for Biotechnology Information. (TIF) [file pbio.2003502.s004.tif]

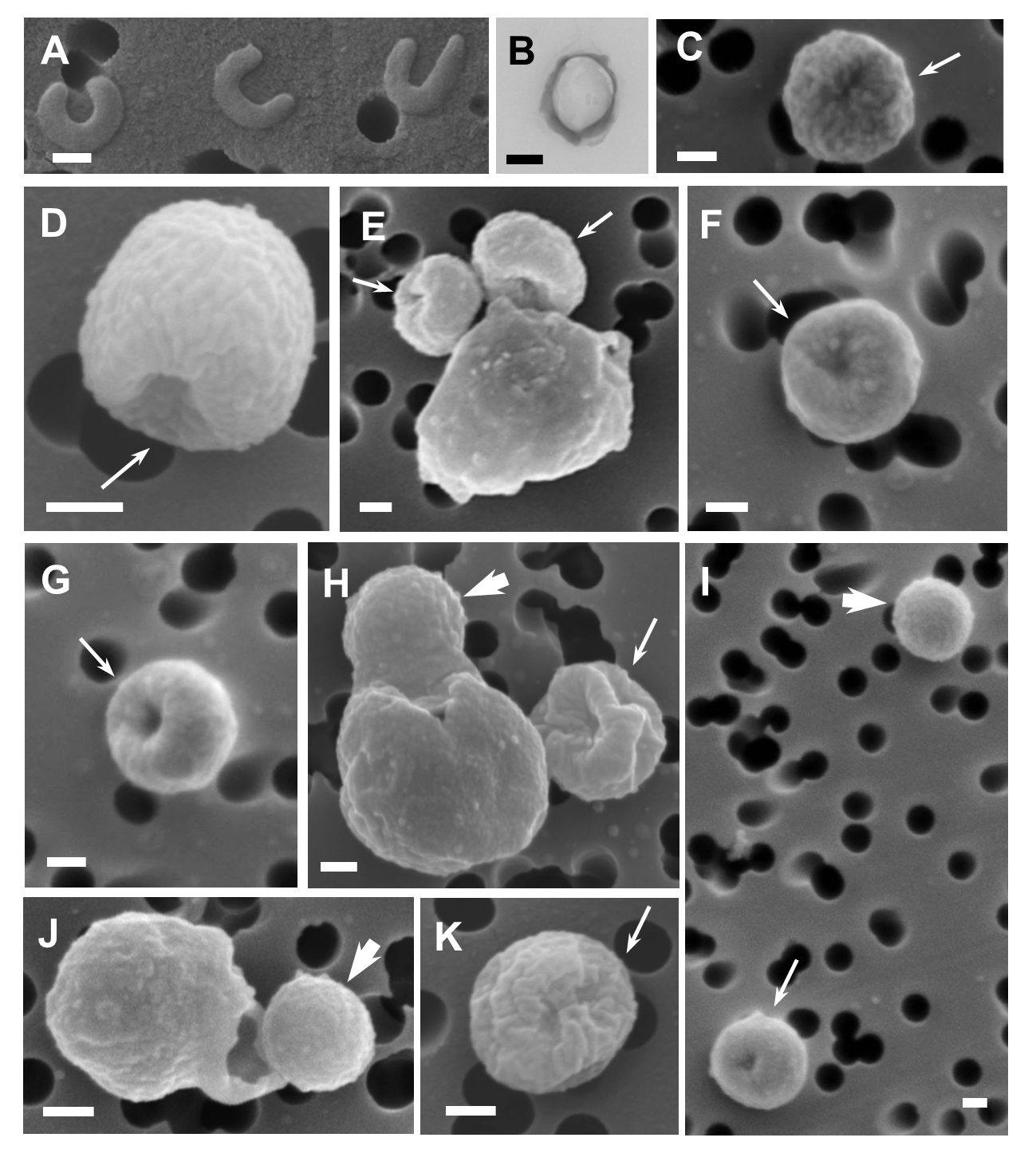

Supplement: S5 Fig — (A) SAR11 alphaproteobacterial cells are presented to compare their morphology with morphology of Prochlorococcus cells. (B) A 0.4 × 0.5–μm body recorded with TEM is most likely a UCYN-A cyanobiont of B. bigelowii, which was lost during deposition of flow-sorted algae onto a grid. (C–K) Ball-shaped intact Prochlorococcus cells (H–J, thick arrows) compared to Prochlorococcus cells with characteristic morphological deformations (thin arrows). In the deformed Prochlorococcus cells, note the depression(s), which transform the Prochlorococcus cells from a ball shape into a doughnut shape. The deformed Prochlorococcus cells were held by B. bigelowii JC142 and presumably separated from them during sorting and dehydration. Scale bar = 0.2 μm. TEM, transmission electron microscopy; UCYN-A, unicellular diazotrophic cyanobacteria group A. (TIF) [file pbio.2003502.s005.tif]

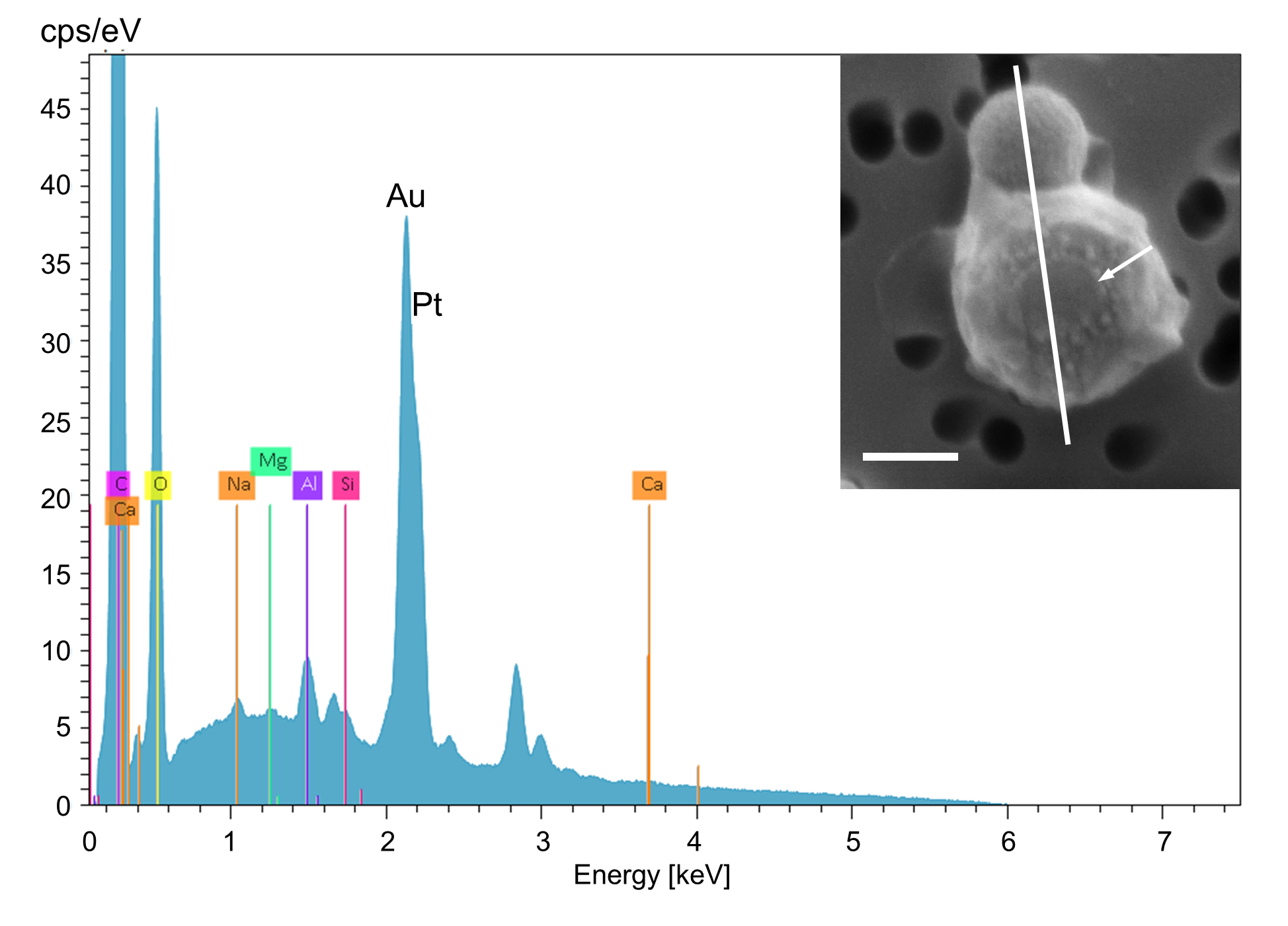

Supplement: S6 Fig — The collected spectrum has distinct peaks of C, N, and O of algal organic materials (polycarbonate support filter contributed only to the C signal) as well as peaks of Au, Pt, and Al originated from the sputtered Au-Pt coating and the aluminium sample stub. The extracellular scale-like investment (arrow) is not calcified because the spectrum showed no detectable Ca. Ca, calcium; SEM, scanning electron microscopy. (TIF) [file pbio.2003502.s006.tif]

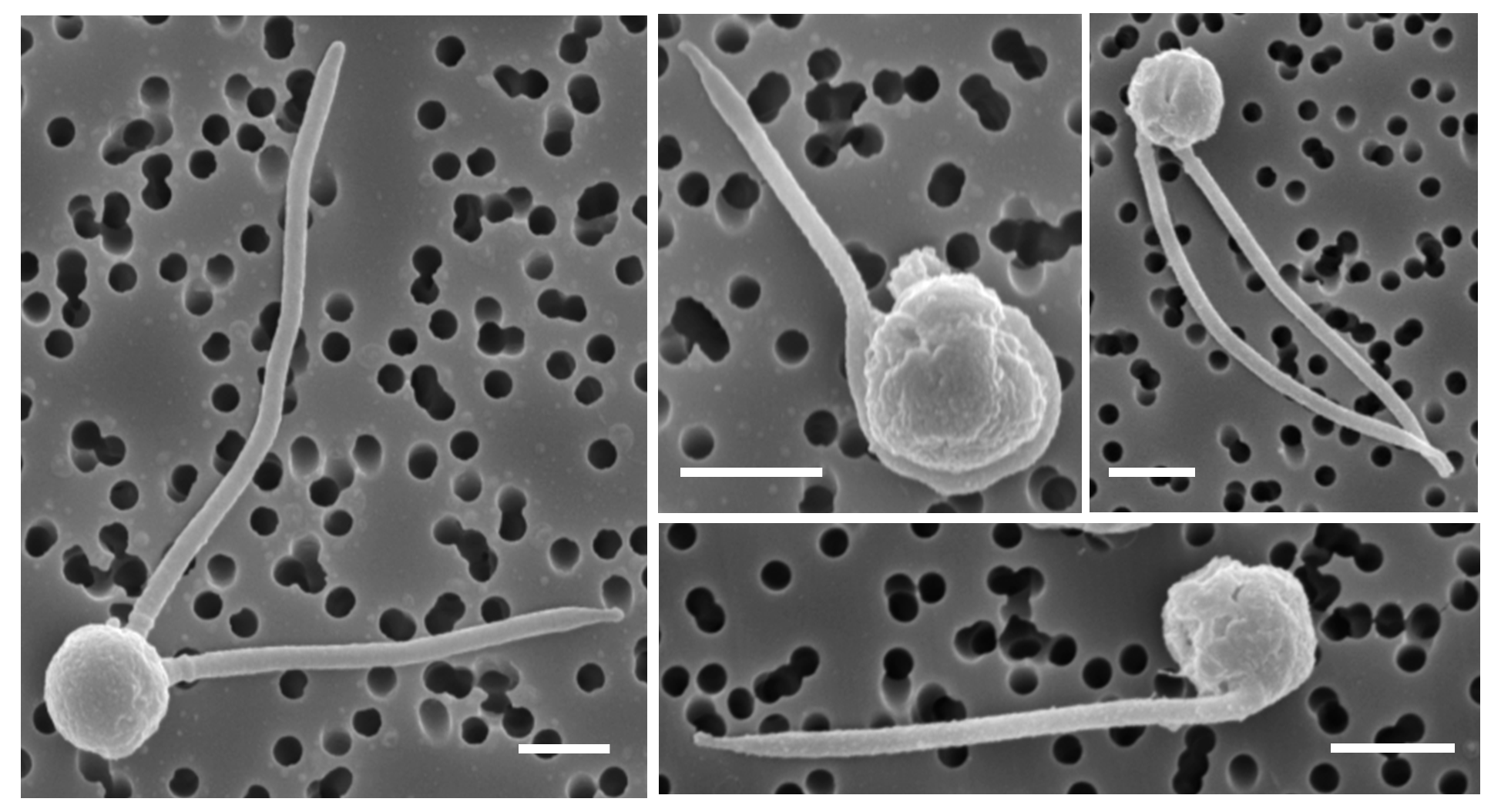

Supplement: S7 Fig — Out of 195 examined cells, only 10 cells had alternative morphology. Note isokont flagella with the distinct basal bodies and pointed tips. Scale bar = 0.5 μm. JC142, the Royal Research Ship “James Cook” cruise number 142; SEM, scanning electron microscopy; PES, plastidic eukaryote small. (TIF) [file pbio.2003502.s007.tif]

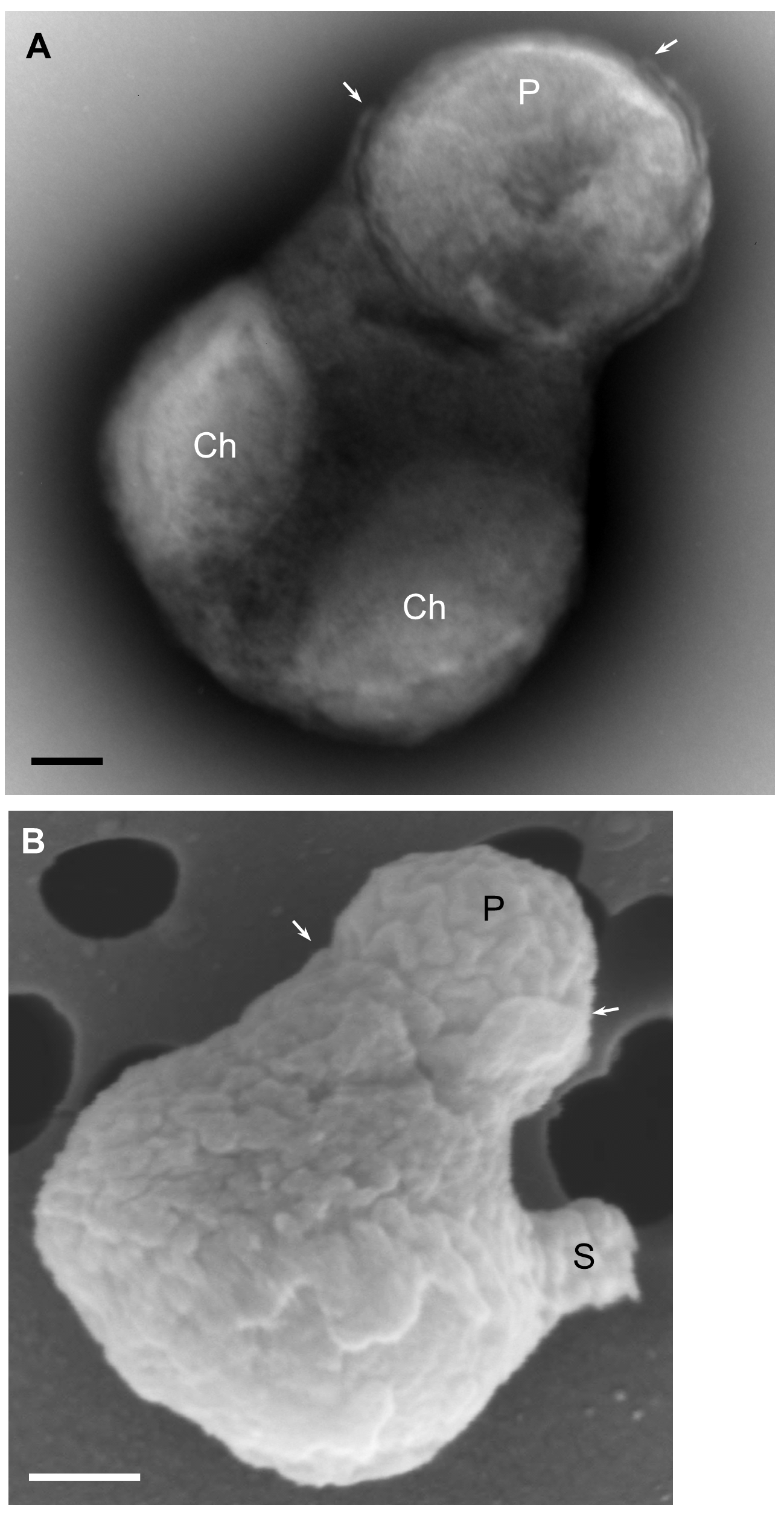

Supplement: S8 Fig — Representative TEM (A) and SEM (B) micrographs show how the Prochlorococcus cell is embraced with the partially open cytostome. Arrows indicate the cytostome edge. Scale bar = 0.2 μm. Ch, chloroplast; P, Prochlorococcus prey; S, cyanobiont; SEM, scanning electron microscopy; TEM, transmission electron microscopy. (TIF) [file pbio.2003502.s008.tif]
